# Renal Cell Carcinoma in the Head and Neck: Case Presentation of a Patient With a Rare Metastatic Pattern

**DOI:** 10.7759/cureus.11894

**Published:** 2020-12-04

**Authors:** Christopher Lenkeit, Julia Bank, Mobeen Shirazi

**Affiliations:** 1 Otolaryngology, McLaren Oakland Hospital, Pontiac, USA; 2 Medicine, Midwestern University Chicago College of Osteopathic Medicine, Downers Grove, USA; 3 Otolaryngology - Head and Neck Surgery, Affiliated Ear Nose and Throat Physicians, Woodstock, USA

**Keywords:** renal cell carcinoma, base of tongue metastasis, trapezius metastasis, head and neck cancer, oral cavity, oropharynx

## Abstract

Renal cell carcinoma is known for its metastatic potential, however, metastasis to the head and neck are rare. We present a 71-year-old man who presented with a palpable tongue mass. The positron-emission tomographic-computed tomographic scan revealed enhancements in the left tongue base, left thyroid, left shoulder musculature, right upper thigh, and right paratracheal mediastinal lymph nodes. Subsequent tongue and trapezius muscle biopsies had immunochemical stains consistent with renal cell carcinoma metastasis. This article discusses an uncommon metastatic pattern of renal cell carcinoma to the tongue and what is the second reported metastasis of renal cell carcinoma to the trapezius muscle.

## Introduction

Renal cell carcinoma (RCC) is an aggressive cancer that presents classically with the triad of hematuria, flank pain, and a palpable abdominal mass. RCC is known to metastasize quickly and is usually asymptomatic until the disease progression is advanced. The most common sites of metastasis include the brain, bone, lung, and liver. RCC metastasis to the head and neck are uncommon, accounting for only 6% of metastasis, most commonly involving the thyroid, parotid gland, and sinuses [1]. Metastasis to the tongue is rare, with a recent study showing a prevalence of less than 0.15% [2]. Metastatic lesions to the trapezius muscle are very rare and have only been confirmed in one other case report [3]. In this report, we describe a 71-year-old asymptomatic patient who presented to the Ear, Nose and Throat (ENT) clinic with a palpable tongue mass. Further imaging and biopsies revealed multiple sites of rare head and neck metastasis 10 years after surgical treatment of primary neoplasm.

## Case presentation

**Figure 1:**
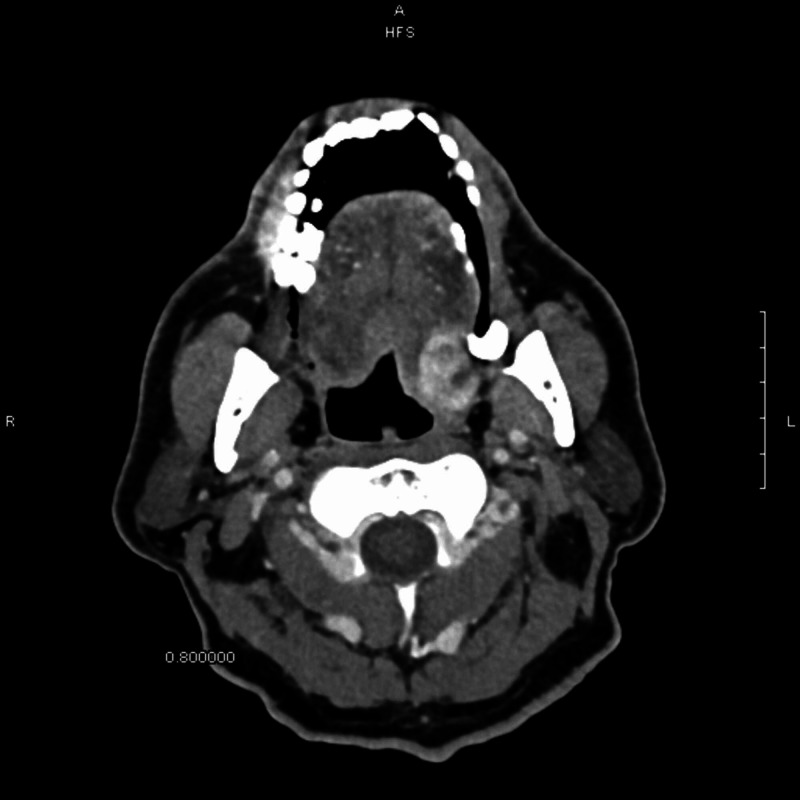
Surveillance head CT showing a suspicious lesion on the left base of tongue.

A 71-year-old male presented to the ENT outpatient clinic with a left posterior tongue mass, found on surveillance computed tomographic (CT) scan (Figure 1). The patient denied any symptoms of dysphagia, taste disturbance, pain, or bleeding. Physical examination revealed a palpable left-sided base of tongue mass that was smooth, non-tender, and non-hemorrhagic. Hypoglossal nerve function was intact and the mass did not restrict tongue mobility or swallowing. There was no palpable cervical lymphadenopathy. The remainder of the head and neck exam was unremarkable. A review of the patient’s history revealed a diagnosis of RCC 10 years prior, with subsequent left-sided nephrectomy. A CT scan of the neck performed at an outside institution revealed an enhancing soft tissue mass along the left posterior tongue base and an enlarged left thyroid lobe.

**Figure 2:**
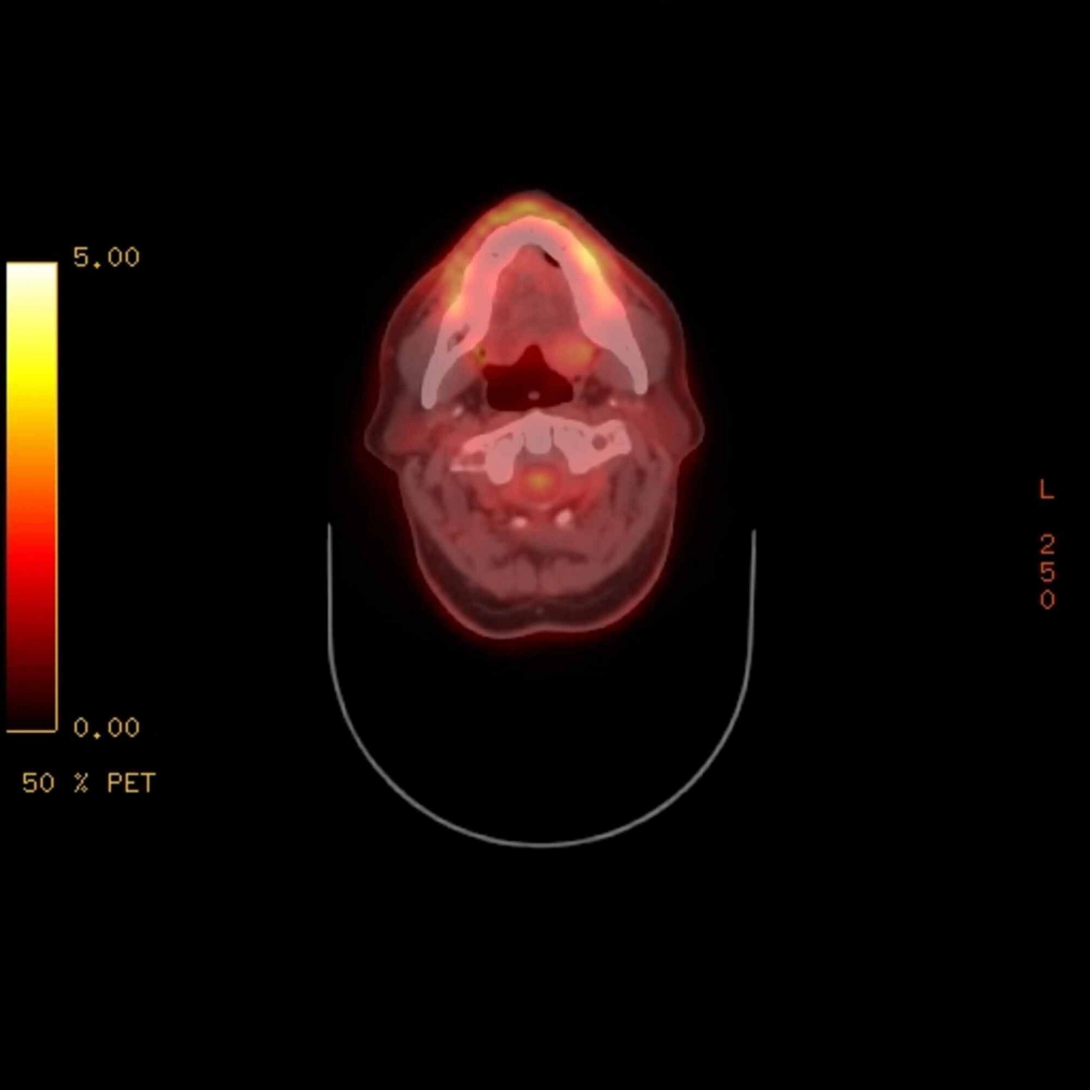
The left posterior tongue mass with increased uptake measuring about 1.5 cm x 0.6 cm (standardized uptake values (SUV) 2.6).

**Figure 3:**
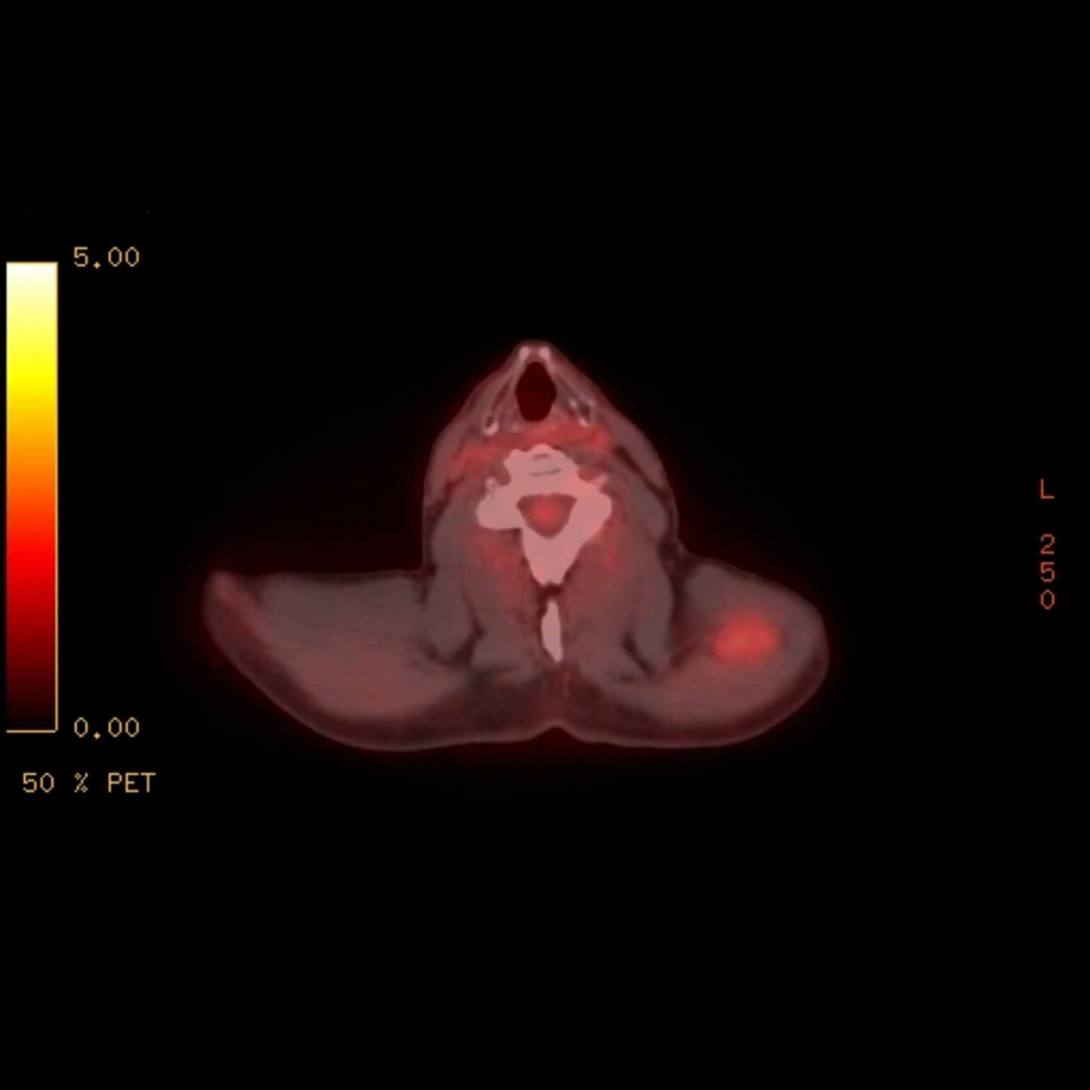
Positron-emission tomographic (PET)-CT scan showing increased uptake in the left trapezius muscle measuring 3.1 cm x 1.9 cm (standardized uptake values (SUV) 2.1).

**Figure 4:**
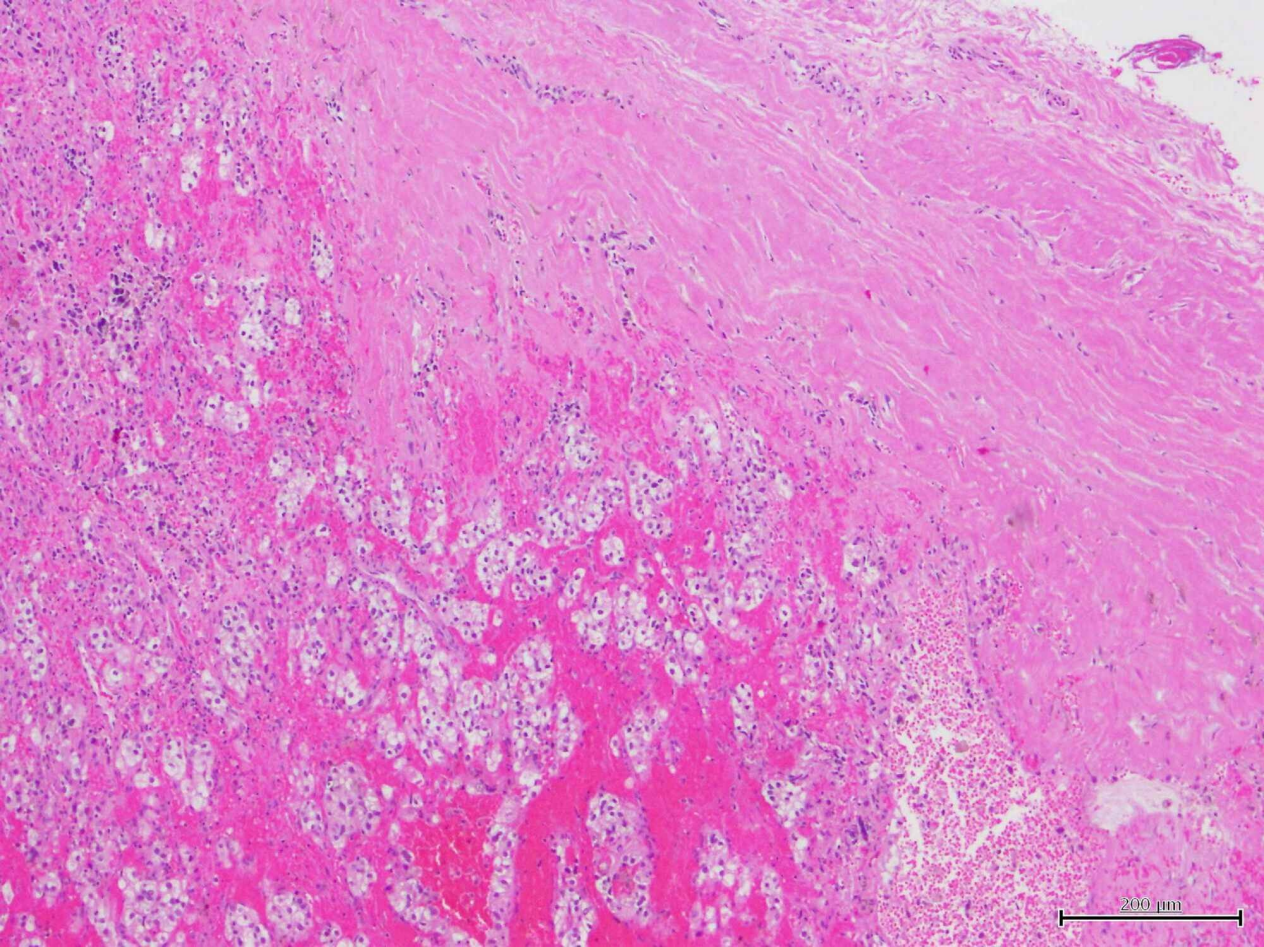
Initial surgical pathology of base of tongue lesion displayed hyperkeratosis of the epithelium and the presence of seromucinous glands appearing to infiltrate the overlying epithelium.

**Figure 5:**
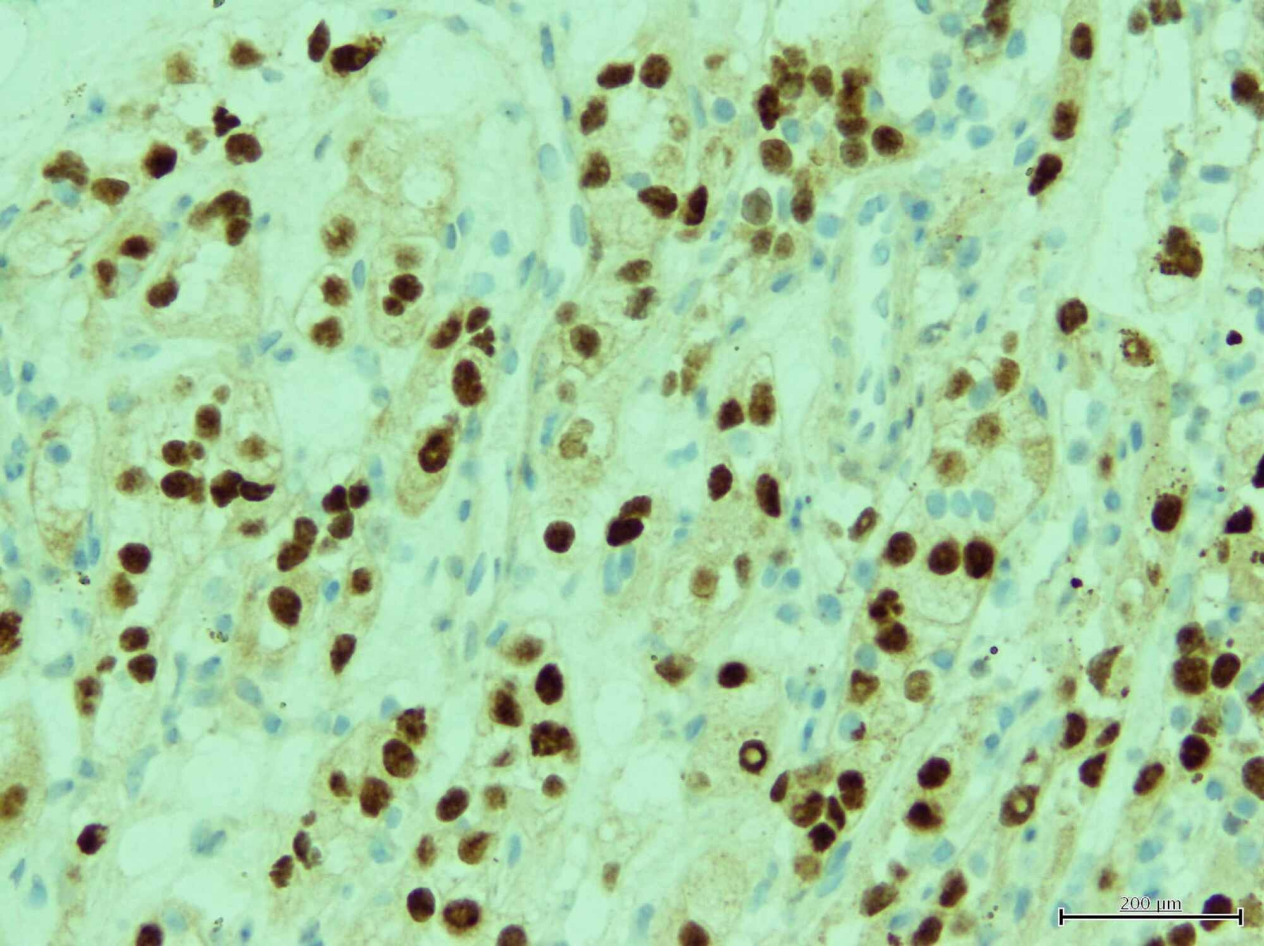
Immunochemical staining for PAX 8 was performed on tongue biopsy confirming renal carcinoma metastasis.

**Figure 6:**
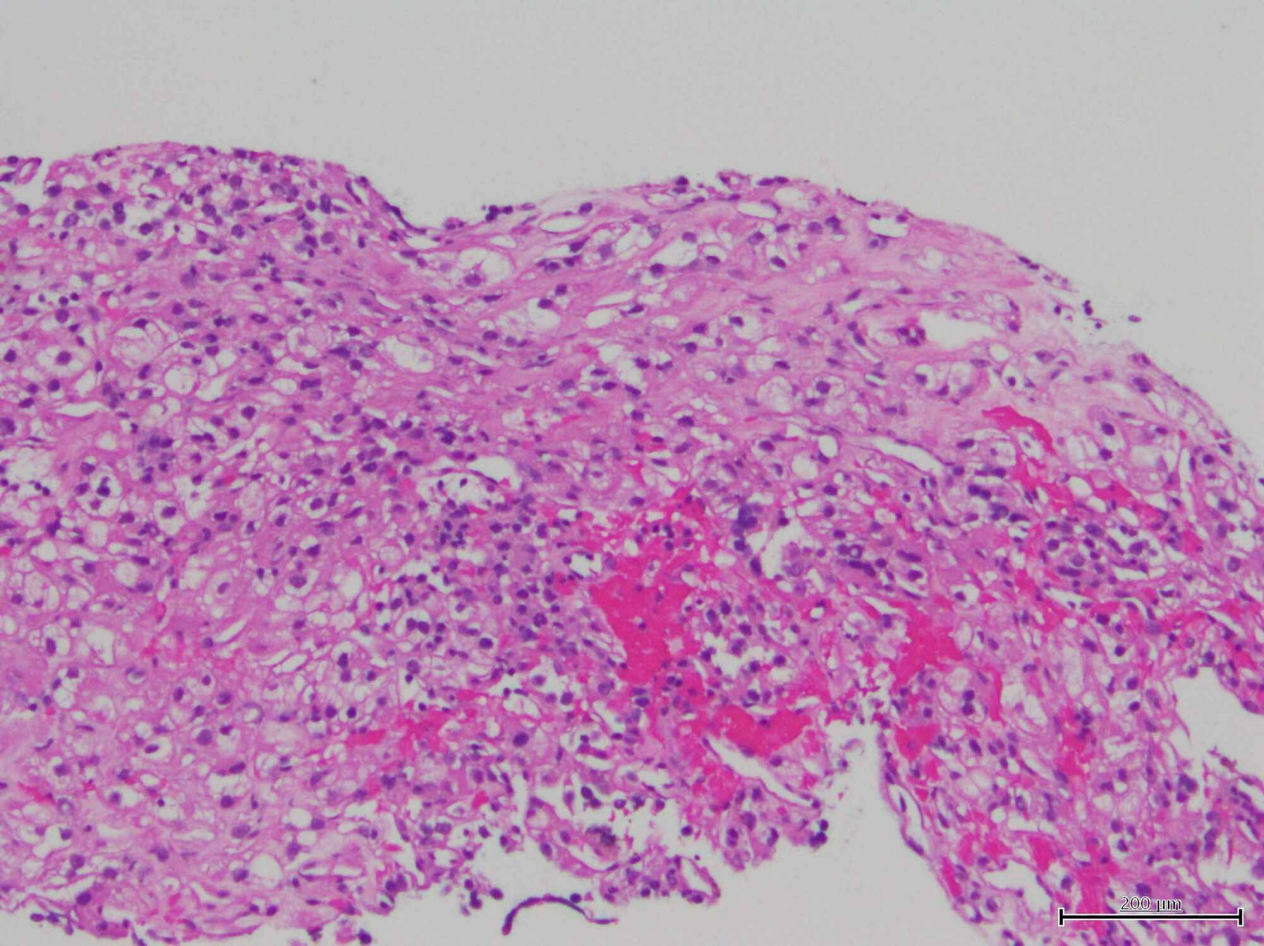
Needle biopsy from left trapezius muscle showing hemorrhagic regions infiltrate by atypical clear cells.

**Figure 7:**
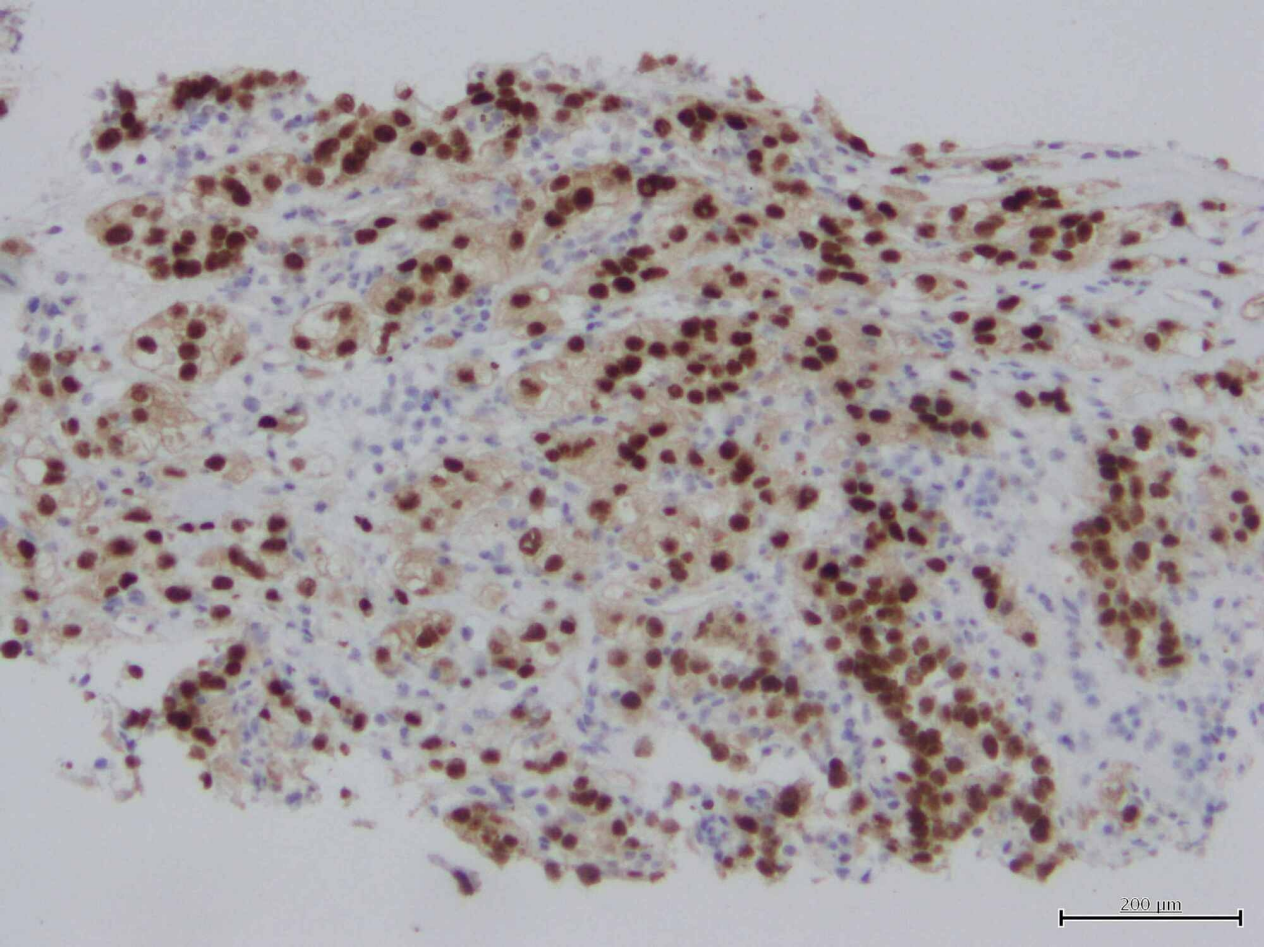
Immunochemical staining for PAX 8 on trapezius muscle biopsy suggestive of renal cell metastasis.

Given the patient’s history of RCC, a positron-emission tomographic (PET)-CT scan was completed from skull base to the thigh for further work-up of the lesion. The scan revealed enhancements in the left tongue base (Figure 2), left thyroid, left shoulder musculature (Figure 3), right upper thigh and right paratracheal mediastinal lymph nodes. Given the patient’s history of RCC and the findings on the PET scan, the decision was made to take the patient to the operating room (OR) for endoscopy under anesthesia with tongue biopsy. Examination in the OR revealed a 1.5 x 1.0 x 0.5-centimeter submucosal smooth mass at the left posterior tongue base. There did not appear to be extension into the supraglottic space. Initial surgical pathology of the base of tongue lesion showed hyperkeratosis of the epithelium and the presence of seromucinous glands appearing to infiltrate the overlying epithelium (Figure 4). Given the difficulty of identifying renal cell carcinoma, immunochemical stains were needed to confirm the diagnosis. Immunochemical staining for PAX8, a marker specific for RCC, was performed on the tongue biopsy. The results showed atypical infiltrates of foamy cells that stained positive for PAX8 (Figure 5). Left trapezius intramuscular mass biopsy showed skeletal muscle with hemorrhagic regions infiltrated by atypical foamy cells (Figure 6). Staining of this biopsy revealed atypical cells that stained positive for PAX8, confirming the diagnosis (Figure 7).

## Discussion

Renal cell carcinoma is the ninth most common cancer worldwide, with a higher incidence in developed countries [4]. There are a wide variety of environmental and genetic risk factors associated with the development of RCC [5]. Common symptoms include a palpable abdominal mass, hematuria, and polycythemia; however, patients are generally asymptomatic until there is widespread, metastatic disease. RCC is an aggressive malignancy with 30% to 40% of patients having metastatic disease at the time of diagnosis [6].

Malignant lesions of the tongue are generally squamous cell carcinomas, however, metastatic lesions to the tongue are rare occurring with an incidence of 0.2% [6]. Breast and lung cancers are the most common malignancies to metastasize to the head and neck. RCC generally spreads hematogenously, most commonly to the brain, lungs, liver, and bone via the inferior vena cava. RCC metastasis to the head and neck are uncommon, accounting for only 6% of metastasis, most commonly involving the thyroid, parotid gland, and sinuses [1]. Despite being an uncommon occurrence, head and neck symptoms were the presenting complaint in 7.5% of patients with metastatic RCC. Recent studies have estimated the incidence of RCC to the tongue specifically to be less than 0.15%, making it a rare manifestation of metastatic RCC [2]. It has also been postulated that the tongue is inhospitable to metastatic cells because it is an environment that must withstand a wide spectrum of temperature, chemical, and mechanical stress [3].

Metastasis to skeletal muscle is very rare, however, they tend to be asymptomatic and skeletal muscle metastasis tends to be microscopic and cannot be regularly identified on gross examination [3]. There are some reported cases of RCC metastasis to skeletal muscle, however, a comprehensive search only revealed one other documented case [3]. Skeletal muscle is a very rare metastatic location possibly due to the secretion of anti-neoplastic factors such as tumor necrosis factor α (TNFα), transforming growth factor beta (TGF-β), and adenosine receptor agonists.

Patients with metastatic RCC are generally treated both surgically and medically. Typically, patients undergo a nephrectomy prior to the initiation of systemic chemotherapy. Median survival is five to 30 months after diagnosis depending on the extent of the patient’s neoplastic disease. Interferon alfa was the former drug of choice, however, newer drugs including cytokines, vascular endothelial growth factor (VEGF) inhibitors, and mammalian target of rapamycin (mTOR) inhibitors are quickly becoming the mainstay treatment options [5]. Despite recent advancements in pharmacologic therapy, surgery remains the treatment of choice for both primary and metastatic lesions [7]. Metastasis to the tongue is a sign of advanced disease. The two-year survival rate is only 22% for patients with oral metastasis. The mean interval from the diagnosis of tongue metastasis to death was 5.8 months [6]. Treatment is palliative and aimed towards patient comfort. Palliative treatment may consist of radiation, though surgical resection is preferred. Options include simple amputation or partial glossectomy with attempts to preserve the function of the tongue.

## Conclusions

RCC is an aggressive malignancy that metastasizes quickly. Head and neck metastasis is uncommon, however, metastasis to the tongue and trapezius muscle is very rare. Surveillance for metastatic lesions must continue for many years after primary diagnosis as evidenced by multiple rare metastatic masses found 10 years after the removal of the primary neoplasm in our patient. Physicians must have a high index of suspicion when evaluating patients with a prior history of any malignancy, including RCC, paying special attention to any new head and neck symptoms.
